# Erythropoietin‐derived peptide ARA290 mediates brain tissue protection through the β‐common receptor in mice with cerebral ischemic stroke

**DOI:** 10.1111/cns.14676

**Published:** 2024-03-15

**Authors:** Rong‐Liang Wang, Zhen‐Hong Yang, Yu‐You Huang, Yue Hu, Yi‐Lin Wang, Feng Yan, Yang‐Min Zheng, Zi‐Ping Han, Jun‐Fen Fan, Zhen Tao, Hai‐Ping Zhao, Si‐Jie Li, Yu‐Min Luo

**Affiliations:** ^1^ Institute of Cerebrovascular Diseases Research and Department of Neurology Xuanwu Hospital of Capital Medical University Beijing China; ^2^ Center of Stroke Beijing Institute for Brain Disorders Beijing China; ^3^ Beijing Key Laboratory of Translational Medicine for Cerebrovascular Diseases Beijing China; ^4^ Emergency Department Xuanwu Hospital of Capital Medical University Beijing China

**Keywords:** ARA290, cerebral ischemia, neuroprotection, β‐Common receptor

## Abstract

**Aim:**

To explore the neuroprotective effects of ARA290 and the role of β‐common receptor (βCR) in a mouse model of middle cerebral artery occlusion (MCAO).

**Methods:**

This study included male C57BL/6J mice that underwent MCAO and reperfusion. The neuroprotective effect of ARA290 on MCAO‐induced brain injury was investigated using neurological function tests (Longa and modified neurological severity score). Cerebral infarction was examined by 2, 3, 5‐triphenyl tetrazolium chloride staining, neuronal apoptosis was assessed by immunofluorescence staining, blood parameters were measured using a flow cytometry‐based automated hematology analyzer, liquid chromatography with tandem mass spectrometry was used to identify the serum metabolomics signature, inflammatory cytokines and liver index were detected by commercially available kits, and the protein levels of the erythropoietin (EPO) receptor and βCR were measured by western blot.

**Results:**

ARA290 exerted a qualitatively similar neuroprotective effect after MCAO as EPO. ARA290 significantly reduced neuronal apoptosis and the level of inflammatory cytokines in the brain tissue. However, ARA290's neuroprotective effect was significantly suppressed following the injection of siRNA against βCR.

**Conclusion:**

ARA290 provided a neuroprotective effect via βCR in cerebral ischemic mice without causing erythropoiesis. This study provides novel insights into the role of ARA290 in ischemic stroke intervention.

## Graphical abstract

ARA290 exerts a neuroprotective action against cerebral ischemic injury by suppressing neuronal apoptosis and inflammatory reactions in mice without motivating splenomegaly and erythropoiesis via the EPO receptor monomer and β‐common receptor.

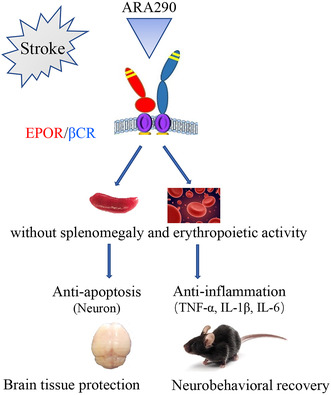

## 1 INTRODUCTION

Over the past two decades, several experimental studies and clinical trials have investigated the neuroprotective function of erythropoietin (EPO) in cerebral ischemic stroke, especially because of its anti‐apoptosis and anti‐inflammatory properties. Unfortunately, its hematopoietic side effects and the unfavorable interactions with recombinant tissue plasminogen activator (rtPA) have limited its clinical application.^1^, ^2^, ^3^ Some studies have demonstrated that non‐hematopoietic variants or analogs of EPO could be potential neuroprotective agents for ischemic stroke therapy.^4^ ARA290, a peptide derived from EPO, is carefully designed to minimize EPO's adverse effects and has garnered attention. Recent reports indicate that ARA290 exerts beneficial effects in multiple preclinical and clinical disease models, such as acute kidney injury, emphysema, diabetic neuropathy, and sarcoidosis‐associated small nerve fiber loss.^5^, ^6^, ^7^, ^8^ Therefore, we hypothesized that ARA290 could be an appropriate candidate against cerebral ischemic‐induced brain injury through its anti‐apoptosis and anti‐inflammatory effects. To our knowledge, only Brines and his colleagues have reported the reduction of brain infarction volume by ARA290 (Helix B, HBP) 24 h after reperfusion in 2008.^9^ There is a shortage of other published studies in this regard. The underlying mechanisms of ARA290's neuroprotective actions and the role of the β‐common receptor (βCR) in ARA290‐mediated protection following cerebral ischemia still require elucidation.

It has been reported that βCR expression in the brain appears on various cell types, such as neurons, astrocytes, microglia, oligodendrocytes, and macrophages.^10^ The role of βCR in the non‐hematopoietic tissue‐protective effects of EPO or its derivatives remains controversial. Brines et al. were the first to demonstrate that EPO's hematopoietic and tissue‐protective activities are distinct and separate.^11^ They proposed that EPO's hematopoietic effects are mediated by the classic EPO receptor homodimer (EPOR/EPOR). In contrast, the tissue‐protective effects are mediated by a hetero‐complex involving the EPOR monomer and βCR (EPOR/βCR) or the βCR subunit alone.^9^ Kanellakis et al. demonstrated that βCR is not implicated in tissue protection and repair.^12^ Furthermore, different opinions exist regarding the direct interaction between the subunits EPOR and βCR. Recently, a study demonstrated that the extracellular regions of EPOR and βCR do not directly interact in the presence of EPO or ARA290.^13^ In contrast, our previous study revealed that EPO treatment immediately after oxygen and glucose deprivation promoted the formation of the EPOR/βCR heterodimer on fetal neural stem cells/neural progenitors.^14^ Given the conditions described, the definite role of βCR in ARA290‐ or EPO‐mediated protection in the ischemic brain remains to be addressed.

Consequently, this study aimed to investigate the potential neuroprotective effects of ARA290 in a mouse model of cerebral ischemia and validate the role of βCR in ARA290‐ or EPO‐mediated neuroprotection.

## 2 METHODS

### 2.1 Animals

Adult male C57BL/6J mice (weighing 22–25 g) were purchased from Vital River Laboratory Animal Technology Co., Ltd. (Beijing, China). The Institutional Animal Care and Use Committee of Capital Medical University approved all animal experiments. The animals were reared under a 12‐h light/dark cycle with free access to water and food. The ambient temperature and humidity were maintained at 22 ± 1°C and 50 ± 10%, respectively.

### 2.2 Mouse model of middle cerebral artery occlusion

Mice underwent middle cerebral artery occlusion (MCAO), as previously described.^15^

### 2.3 Animal grouping and drug administration

ARA290 (Cibinetide) was obtained from MedChem Express (Cat No: HY‐P0168, USA). EPO was obtained from Shenyang Sunshine Pharmaceutical Co. Ltd. (Shenyang, China).

Section 1: To investigate the neuroprotective effect of ARA290, different concentrations and frequencies of ARA290 administration were employed for treating the mice. Each group consisted of 10 mice (total number = 40) subjected to 2, 3, 5‐triphenyltetrazolium chloride (TTC) staining 7 days after stroke. The mice were randomly categorized into four groups: (1) MCAO group, mice underwent MCAO and received saline treatment; (2) 30, qd group, mice underwent MCAO and were administered ARA290 at a dose of 30 μg/kg once a day; (3) 30, bid group, mice underwent MCAO and were administered ARA290 at a dose of 30 μg/kg twice a day; and (4) 100, qd group, mice underwent MCAO and were administered ARA290 at a dose of 100 μg/kg once a day.

Section 2: To verify if ARA290 treatment exerts a neuroprotective effect similar to EPO following cerebral ischemic injury without causing erythropoiesis. Each group consisted of 12 mice (total number = 48) used for TTC staining and hematologic examination. Splenic weight and length were recorded 7 days post‐stroke, and brain tissue loss was tested 14 days post‐stroke. Neurobehavioral evaluation was performed at 1, 3, 5, 7, 11, and 14 days post‐stroke. The mice were randomly classified into four groups: (1) sham‐operated group (Sham), (2) vehicle group (MCAO), (3) EPO group (EPO), and (4) ARA290 group (ARA290). The same volume (100 μL) of the saline vehicle, EPO (5000 U/kg, every other day), or ARA290 (30 μg/kg, bid), was intraperitoneally injected at the beginning of reperfusion after the MCAO surgery.

Section 3: To confirm whether βCR mediates the neuroprotective effects of ARA290 or EPO following MCAO surgery. We evaluated brain infarction volume (7 days after MCAO) and tissue loss volume (14 days after MCAO) using 40 mice randomly categorized into four groups (*n* = 10 each): (1) MCAO + negative control (NC) group, (2) ARA290 + NC group, (3) MCAO + βCR siRNA group, and (4) ARA290 + βCR siRNA group. In another set of experiments, 20 mice were classified into four groups (*n* = 5 each): (1) MCAO + NC group, (2) EPO + NC group, (3) MCAO + βCR siRNA group, and (4) EPO + βCR siRNA group. βCR siRNA or NC was administered by right intracerebroventricular injection immediately after reperfusion. Subsequently, the same volume (100 μL) of the saline vehicle, EPO (5000 U/kg, every other day), or ARA290 (30 μg/kg, bid) was intraperitoneally injected at the beginning of reperfusion after the MCAO surgery.

Section 4: To evaluate the time course of EPOR and βCR protein levels in ischemic brain tissue, six mice were used in each group (total number = 36) to perform TTC staining and western blot analysis at 0.5, 1, 3, 7, and 14 days post‐stroke. The mice were randomly categorized into six groups: (1) sham‐operated group (Sham), (2) MCAO 0.5d, (3) MCAO 1d, (4) MCAO 3d, (5) MCAO 7d, and (6) MCAO 14d.

### 2.4 Transfection of siRNA into the mouse brain

The efficiency of siRNA against βCR in cerebral ischemic mice was first tested. Intracerebroventricular injection of siRNA (GenePharma Co., Ltd., Shanghai, China) was performed 3 days before MCAO surgery, as previously described.^16^ The target sequences were 5′‐GCA AAT TTC AGG TGA ATT TCG‐3′ (βCR siRNA, Catalog number: 201219AZ) and 5′‐TTC TCC GAA CGT GTC ACG T‐3′ (NC, Catalog number: K21YZ).

### 2.5 Neurobehavioral evaluation

Longa tests and modified neurological severity scores (mNSS) were employed to evaluate motor, balance, sensory, and reflex functions daily for 7 days, beginning 1 day before MCAO surgery. The mNSS score ranges from 0 to 14; the higher the score, the more severe the neurological deficit.

### 2.6 Histological assessment

The percentage of the brain infarction volume and tissue loss volume were assessed as previously described.^17^

### 2.7 Immunofluorescence staining and terminal deoxynucleotidyl transferase dUTP nick end labeling assay

Brain sections were processed for immunofluorescent labeling and terminal deoxynucleotidyl transferase dUTP nick end labeling (TUNEL) assays to assess the level of neuronal apoptosis, as previously described.^15^

### 2.8 Metabolomics study

Liquid chromatographic separation for plasma (*n* = 6) was accomplished using the Q Exactive System (Thermo Scientific™) with a Waters ACQUITY UPLC BEH C8 (1.7 μm, 2.1 mm × 100 mm) column, and mass spectrometry was performed on the Thermo Scientific™ Dionex™ ΜltiMate™ 3000 Rapid Separation LC. Data pretreatment procedures were performed using Progenesis QI 2.3 software (Waters Corporation, MA, USA). Open database sources were used to identify metabolic pathways, including KEGG, MetaboAnalyst, the Human Metabolome Database, and METLIN.

### 2.9 Measurement of inflammatory cytokines in mice brain

Mouse brain samples were homogenized in a normal saline solution on ice at 1 and 7 days post‐MCAO, and the supernatants were subsequently collected by centrifugation. The concentrations of inflammatory cytokines (TNF‐α, IL‐1β, and IL‐6) were assessed using mouse TNF‐α, IL‐1β, and IL‐6 ELISA kits (Neobioscience Technology Co., Ltd, Shenzhen, China) according to the manufacturer's protocol.

### 2.10 Western blot analysis

Western blot analysis was performed as previously described,^15^ using primary antibodies: anti‐EPOR (1:1000, orb10601, Biorbyt, Cambridge, UK), anti‐βCR (1:500, sc‐393,281, Santa Cruz, CA, USA), and anti‐β‐actin (1:1000, sc‐1616, Santa Cruz, CA, USA).

### 2.11 Measurement of erythropoietic effects and serum alanine aminotransferase, aspartate aminotransferase, and lactate dehydrogenase in mice

Blood parameters were measured using a flow cytometry‐based automated hematology analyzer (Mindray BC‐5000Vet; Mindray Animal Medical Technology Co., Ltd, Shenzhen, China) 7 days after MCAO. The spleen was isolated on day 7 postoperatively, and the spleen weight and length were measured to evaluate splenomegaly, indicating the degree of erythropoietic stress. The serum alanine aminotransferase (ALT), aspartate aminotransferase (AST), and lactate dehydrogenase (LDH) levels were determined using kits (Nanjing Jiancheng Bioengineering Institute, Jiangsu, China) 14 days after MCAO.

### 2.12 Statistical analysis

Results are expressed as the mean ± standard error of the mean (SEM). All statistical analyses were performed using GraphPad Prism version 7.0 (GraphPad Software Inc., CA, USA). The D'Agostino and Pearson omnibus normality test was used to analyze the normality of the data, which was found to be normally distributed. Data from the two groups were analyzed using the Student's *t*‐test (for single comparisons) or one‐way analysis of variance (ANOVA), followed by Tukey's post‐hoc test. Differences in means across multiple groups were analyzed using a two‐way repeated‐measures ANOVA, followed by Tukey's multiple comparison test. The results were considered statistically significant at *p* ≤ 0.05.

## 3 RESULTS

### 3.1 ARA290 exerts a similar neuroprotective effect as EPO in cerebral ischemic mice

**FIGURE 1:**
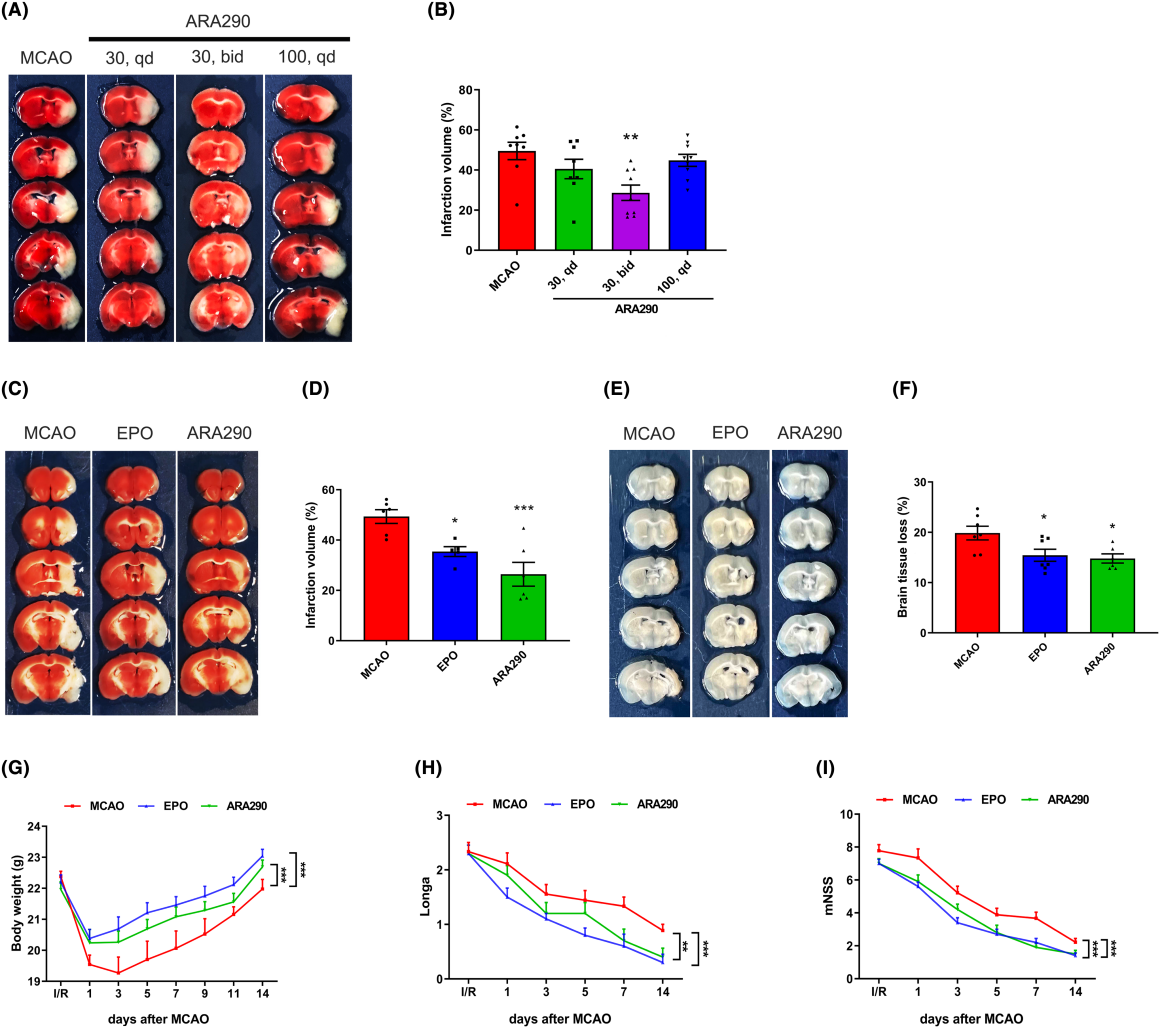
ARA290 exerts a similar neuroprotective effect as erythropoietin (EPO) in cerebral ischemic mice. (A) Representative coronal sections stained with 2, 3, 5‐triphenyl‐tetrazolium chloride (TTC), showing the size of the infarction volume; (B) Schematic diagram of the percentage of brain infarction in different groups at 7 days after middle cerebral artery occlusion (MCAO). ARA290 was injected intraperitoneally (i.p.) at the beginning of reperfusion after the MCAO surgery. 30, qd: administered with ARA290 at doses of 30 μg/kg once daily; 30, bid: administered with ARA290 at doses of 30 μg/kg twice daily; 100, qd: administered with ARA290 at doses of 100 μg/kg once daily. Data are presented as mean ± SEM, *n* = 8–9 per group. **p < 0.01, 30 bid group vs. MCAO group. (C) Representative coronal sections stained with TTC, and (D) a schematic diagram illustrating the percentage of brain infarction in different groups at 7 days after MCAO. Data are presented as mean ± SEM, *n* = 5–6 per group. *p < 0.05, EPO group vs. MCAO group; ***p < 0.001, ARA290 group vs. MCAO group. (E) Representative coronal sections and (F) a schematic diagram of the percentage of brain tissue loss in the different groups 14 days after MCAO. Data are presented as mean ± SEM, *n* = 6–7 per group. *p < 0.05, EPO group vs. MCAO group; *p < 0.05, ARA290 group vs. MCAO group. (G) Body weight in different groups after MCAO. (H, I) Longa test and modified neurological severity scores were evaluated in different groups. Data are presented as mean ± SEM, *n* = 9–10 per group. ***p < 0.001, EPO group vs. MCAO group; **p < 0.01, ARA290 group vs. MCAO group; ***p < 0.001, ARA290 group vs. MCAO group.

The TTC result demonstrated that mice in the 30 bid (30 μg/kg, bid) group had significantly decreased brain infarction volume compared with the MCAO group (*p* < 0.005, Figure 1A,B). Furthermore, ARA290 (30 μg/kg, bid) also exerted protective effects on brain tissue (*p* < 0.001, Figure 1C–F), and no significant difference was observed between the ARA290 and the EPO groups.

Body weight was consistent with the results of brain infarction and brain tissue loss in each group. ARA290 and EPO treatment improved the excessive weight loss after MCAO (*p* < 0.001; Figure 1G). Neurobehavioral tests indicated that ARA290 treatment significantly improved neurological function recovery after MCAO surgery, as demonstrated by lower Longa and mNSS scores than in the vehicle‐treated group following MCAO (*p* < 0.001, Figure 1H,I).

### 3.2 ARA290 attenuates neuronal apoptosis and inflammation in the per‐infarct region after cerebral ischemia

**FIGURE 2:**
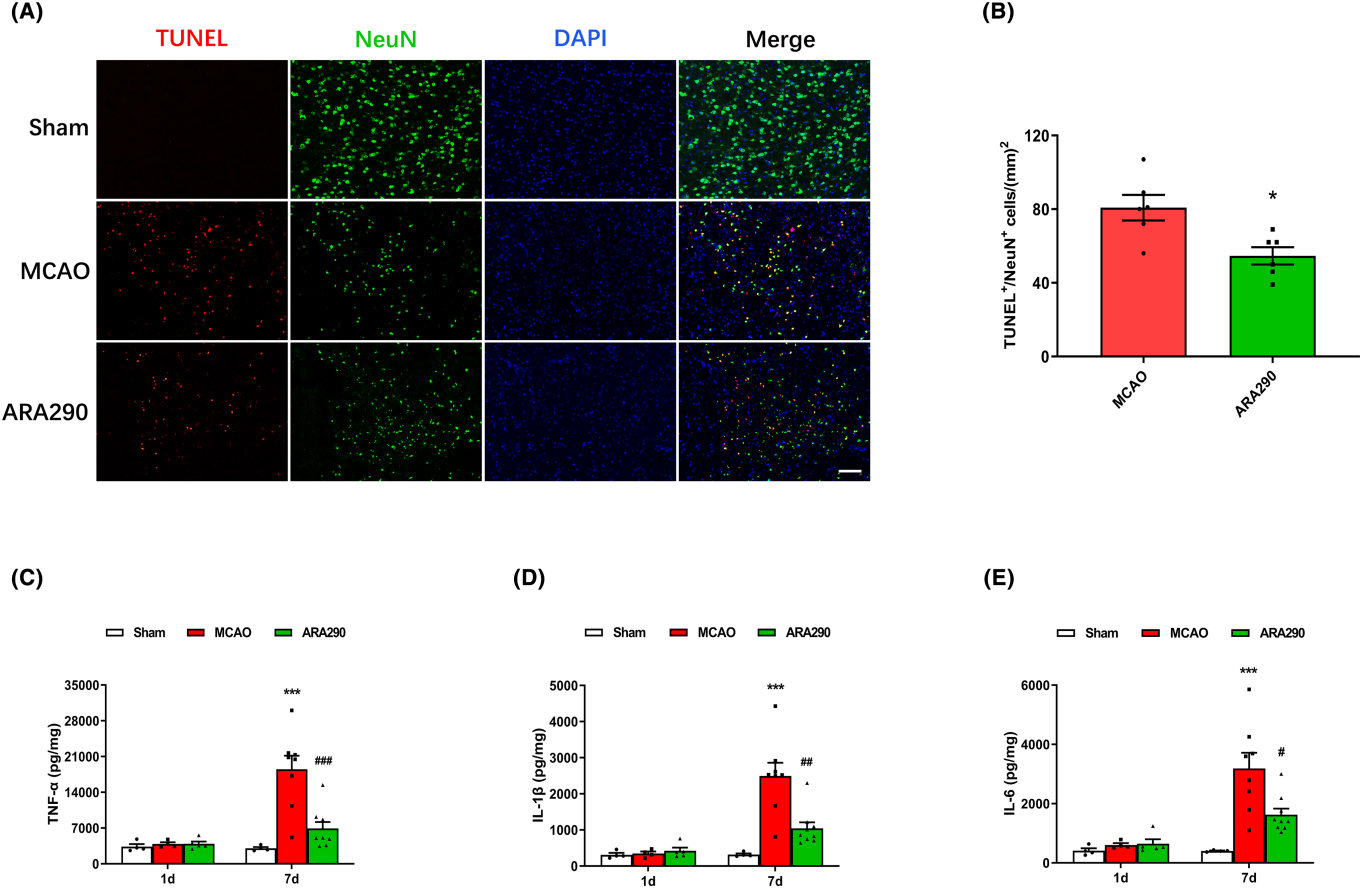
ARA290 attenuates neuronal apoptosis and inflammation in the per‐infarct region after cerebral ischemia. (A) Photomicrographs displaying terminal deoxynucleotidyl transferase dUTP nick end labeling (TUNEL) (red) and NeuN (green) double‐positive cells in the peripheral area of brain infarction in different groups 7 days after middle cerebral artery occlusion (MCAO). DAPI (blue) indicates the cell nuclei. Scale bar, 50 μm. (B) Quantification of TUNEL^+^/NeuN^+^ colabeling cells in different groups. Data are presented as mean ± SEM, *n* = 6 per group. *p < 0.05, ARA290 group vs. MCAO group. The quantification of TNF‐α (C), IL‐1β (D), and IL‐6 (E) expression in the Sham, MCAO, and ARA290 treatment mouse brain at 1 and 7 days post‐MCAO. Data are presented as mean ± SEM, *n* = 4–9 per group. ***p < 0.001, MCAO group vs. Sham group; ^#^ *p* < 0.05, ^##^ *p* < 0.01, ^###^ *p* < 0.001, ARA290 group vs. MCAO group.

Immunofluorescence staining revealed a significant increase in TUNEL/NeuN double‐labeled cells in the MCAO group 7 days after MCAO surgery (Figure 2A). Notably, ARA290 treatment ameliorated neuronal apoptosis compared to that in the MCAO group, potentially contributing to its role in reducing brain infarction (*p* < 0.05, Figure 2B).

The ELISA results showed no statistically significant difference in inflammatory factors (TNF‐α, IL‐1β, and IL‐6) among the sham, MCAO, and ARA290 groups 1 day after MCAO (Figure 2C–E). However, 7 days after cerebral ischemia, compared with the sham group, the findings revealed a significant increase in the levels of inflammatory factors in the MCAO group (*p* < 0.001, Figure 2C–E). The inflammatory factors increased significantly in the ARA290 group compared to the MCAO group (*p* < 0.05, Figure 2C–E).

### 3.3 ARA290 produces a metabolomics signature in cerebral ischemic mice

**FIGURE 3:**
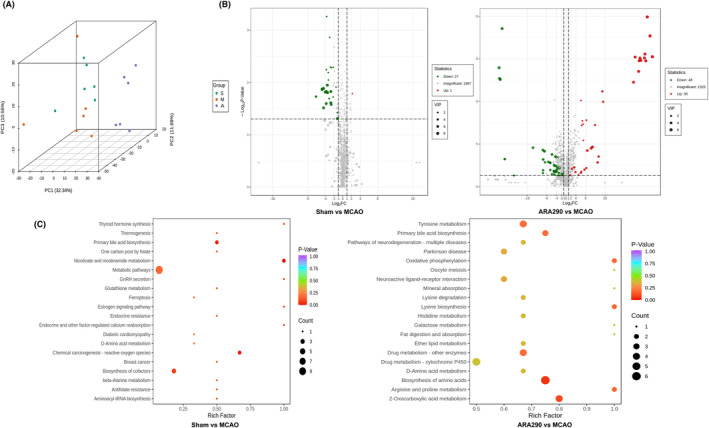
ARA290 produces a metabolomic signature in middle cerebral artery occlusion (MCAO) mice. (A) Principal Component Analysis (PCA) scores among the Sham, MCAO, and ARA290 groups. (B) Volcano plots display all metabolite alterations in the plasma of mice between groups. (C) Metabolic pathway enrichment analysis of differential metabolites in the plasma of mice, *n* = 5 per group. All matched pathways are displayed as circles; the colors of the circles are based on the pathways' *p*‐value, and the size is based on the impact value. Redder and larger circles indicate lower *p*‐values and greater count numbers.

Metabolomic detection can monitor metabolite levels in vivo in real‐time and reflect the overall law of endogenous metabolites and dynamic changes in metabolites in organisms. To investigate whether ARA290 produces a serum metabolomics signature, liquid chromatography with tandem mass spectrometry (LC–MS/MS) was carried out 7 days after mice were subjected to MCAO. After peak alignment and removing missing values, LC–MS/MS detected 1609 metabolites from all 18 plasma samples using nontargeted metabolomic MS analysis. The Principal Component Analysis model was used to characterize the metabolic disturbances. Overall, 176 metabolites exhibited significant differences between the MCAO and sham groups, and 83 metabolites showed considerable differences between the ARA290 and MCAO groups. Metabolites with variable importance in the projection values >1.0 were considered potential differential metabolites (Figure 3A,B).

We compared the Sham, MCAO, and ARA290 groups to identify and characterize specific metabolites and metabolic pathways. Different metabolites were screened based on their differential expression levels (Figure 3B). The primary differential metabolic pathways between the MCAO and sham groups involved thyroid hormone metabolism, thermogenesis, a one‐carbon pool of folate, and primary bile acid biosynthesis pathways (Figure 3C). ARA290 reversed brain metabolic deviations in stroke by significantly upregulating the levels of valdecoxib, 2‐hydroxy‐4‐trifluoromethyl benzoic acid, 5′‐phosphoribosyl‐N‐formylglycinamide, aspartyl hydroxyproline, and the primary differential metabolic pathways involving tyrosine metabolism, primary bile acid biosynthesis, pathways of neurodegeneration, multiple diseases, and oxidative phosphorylation pathways (Figure 3C). In conclusion, the primary differential metabolites of ARA290 were mostly related to the pathophysiology of the vascular endothelium and oxidative stress, which may be relevant to its anti‐apoptotic and anti‐inflammatory properties.

### 3.4 ARA290 abolishes the erythropoietic activity of EPO without potential safety issues

**FIGURE 4:**
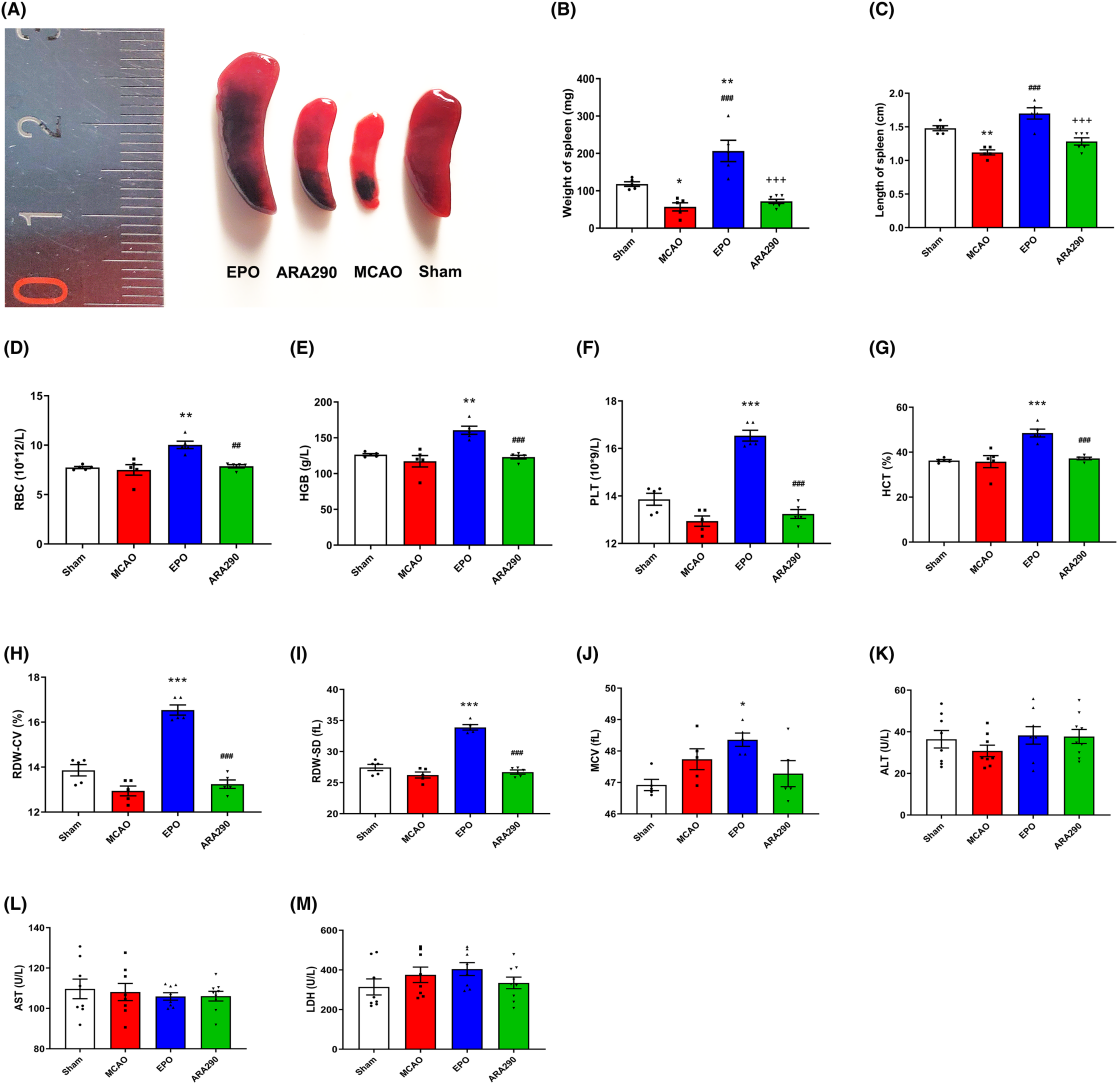
ARA290 abolishes the erythropoietic activity of erythropoietin (EPO) without potential safety issues. (A) Respective representative photographs of spleens from different groups 7 days after middle cerebral artery occlusion (MCAO) surgery. Histograms showing the spleen's weight (B) and length (C) in different groups. Data are presented as mean ± SEM, *n* = 5–7 per group. *p < 0.05, **p < 0.01, MCAO group vs. Sham group; **p < 0.05, EPO group vs. Sham group; ^###^ *p* < 0.001, EPO group vs. MCAO group; ^+++^ *p* < 0.001, ARA290 group vs. EPO group. Quantification of the blood parameters of RBC (D), HGB (E), PLT (F), HCT (G), RDW‐CV (H), RDW‐SD (I), and MCV (J) from different groups 7 days after MCAO surgery. Data are presented as mean ± SEM, *n* = 5 per group. *p < 0.05, **p < 0.01, ***p < 0.001, EPO group vs. Sham group; ^##^ *p* < 0.01, ^###^ *p* < 0.001, ARA290 group vs. EPO group. HCT, hematocrit; HGB, hemoglobin; MCV, mean corpuscular volume; PLT, platelet; RBC, red blood cell; RDW‐CV, coefficient variation of red blood cell volume distribution width; RDW‐SD, standard deviation in red cell distribution width. (K–M) The serum alanine aminotransferase (ALT), aspartate aminotransferase (AST), and lactate dehydrogenase (LDH) levels were determined by commercial reagent kits 14 days after MCAO surgery. Data are presented as mean ± SEM, *n* = 8–9 per group.

Our results revealed a significant decrease in both the weight and length of the spleen in the vehicle group compared to those in the sham group 7 days after MCAO (*p* < 0.05, Figure 4A–C), indicating ischemic stroke‐induced splenic atrophy. Compared to the vehicle‐treated group, EPO‐treated mice exhibited a significant increase in both the weight and length of the spleen (*p* < 0.001, Figure 4A–C). In contrast, the ARA290‐treated group demonstrated significantly lower spleen weight and length than the EPO‐treated group (*p* < 0.001, Figure 4A–C). In summary, the weight and length of the spleen in the ARA290 group remained unchanged.

Blood tests indicated that EPO treatment significantly increased various blood parameters such as red blood cell count, hemoglobin levels, platelet count, hematocrit, coefficient variation of red blood cell volume distribution width (RDW‐CV), standard deviation in red cell distribution width (RDW‐SD), and mean corpuscular volume (MCV) compared to those in the sham group (*p* < 0.05, Figure 4D–J). As anticipated, none of the erythropoietic indicators significantly changed in the ARA290‐treated group compared to the sham group (Figure 4D–J).To assess the effect of ARA290 on liver function and its toxicity, the levels of serum biomarkers AST, ALT, and LDH were tested 14 days after MCAO. There was no obvious difference among the different thread groups (Figure 4K–M). The results revealed that ARA290 is safe for treating MCAO‐induced cerebral ischemic injury.

### 3.5 βCR is essential for ARA290, providing a neuroprotective effect against cerebral ischemic stroke

**FIGURE 5:**
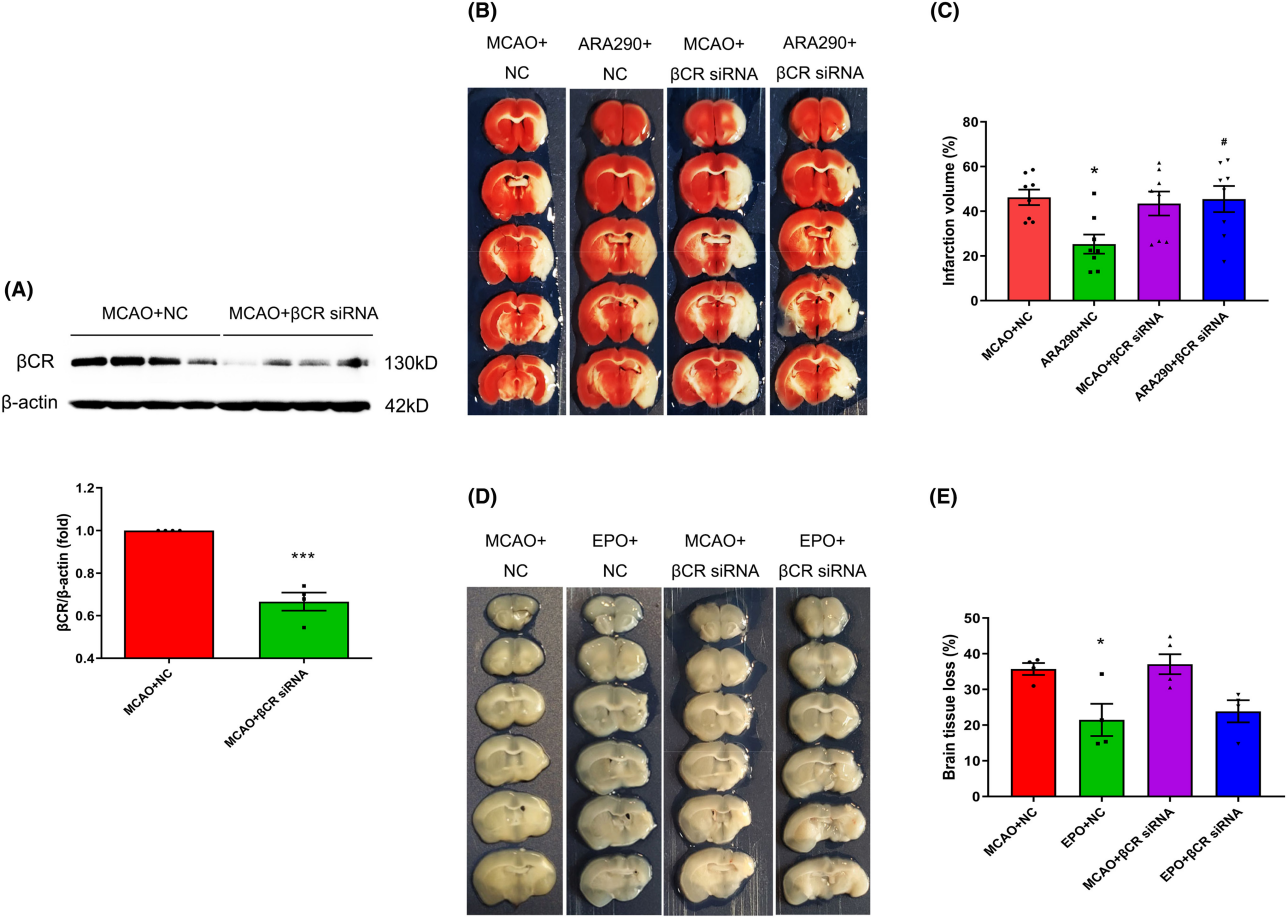
β‐Common receptor (βCR) is essential in the neuroprotective effect of ARA290 against cerebral ischemic stroke. (A) Western blot analysis shows the efficiency of βCR siRNA on the expression of βCR in brain tissue 1 day after middle cerebral artery occlusion (MCAO) surgery. β‐Actin was used as a control to normalize protein expression. Data are presented as mean ± SEM, *n* = 4 per group. ***p < 0.001, MCAO + βCR siRNA group vs. MCAO + negative control (NC) group. (B) Representative coronal sections stained with 2, 3, 5‐triphenyltetrazolium chloride (TTC) and (C) a schematic diagram of the percentage of brain infarction in the different groups 7 days after MCAO. Data are presented as mean ± SEM, *n* = 8 per group. *p < 0.05, ARA290 + NC group vs. MCAO + NC group; ^#^ *p* < 0.05, ARA290 + βCR siRNA group vs. ARA290 + NC group. (D) Representative coronal sections and (E) schematic diagram of the percentage of brain tissue loss in the different groups 14 days after MCAO. Data are presented as mean ± SEM, *n* = 4–5 per group. *p < 0.05, erythropoietin (EPO) + NC group vs. MCAO + NC group.

As anticipated, the western blot demonstrated that βCR siRNA significantly suppressed βCR expression at day 1 after MCAO (*p* < 0.001; Figure 5A). Consistent with our previous study, TTC staining results indicated that ARA290 treatment significantly reduced the percentage of brain infarction volume compared to MCAO 7 days after MCAO (*p* < 0.05, Figure 5B,C). However, when ARA290 was combined with βCR siRNA treatment, it attenuated the tissue protective effect of ARA290 treatment alone (*p* < 0.05, Figure 5B,C). In another part of the experiment, EPO treatment significantly decreased the percentage of brain tissue loss volume compared to the vehicle treatment (MCAO + NC) at day 14 after MCAO (*p* < 0.05, Figure 5D,E). However, the tissue‐protective effect of EPO disappeared when EPO was combined with the βCR siRNA treatment (Figure 5D,E). These results demonstrate that inhibiting βCR expression abolished ARA290‐ and EPO‐conferred neuroprotection from MCAO‐induced brain infarction and brain tissue loss.

### 3.6 Temporal changes in βCR protein levels are related to degrees of brain injury in mice after cerebral ischemia

**FIGURE 6:**
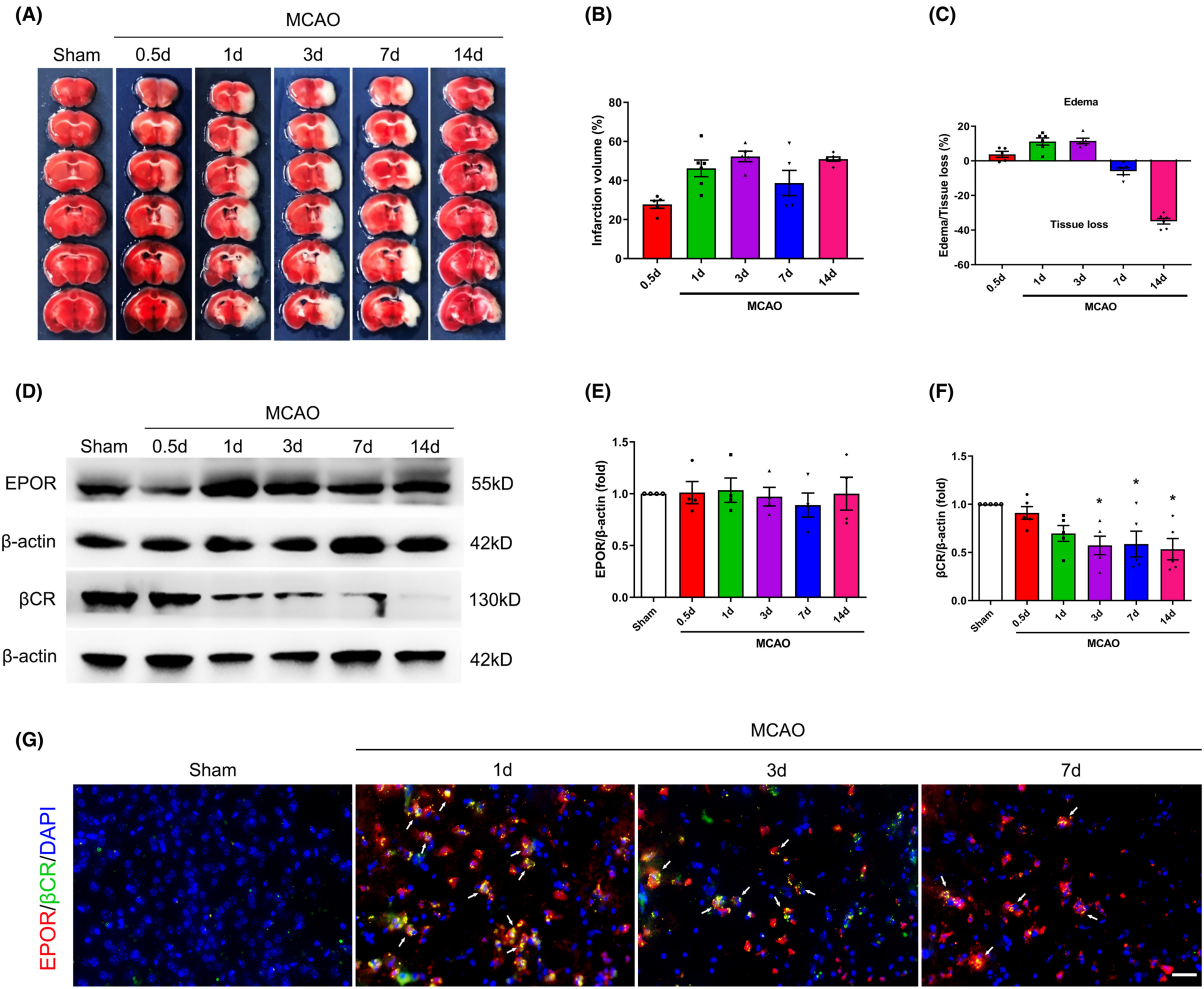
Temporal changes in the β‐common receptor **(**βCR) protein levels are related to brain injury degrees in mice after cerebral ischemia. (A) Representative coronal sections images of 2, 3, 5‐triphenyltetrazolium chloride (TTC) staining and the schematic diagram of the percentage of brain infarction volume (B), brain edema volume, or brain tissue loss volume (C) in different groups at 0.5, 1, 3, 7, and 14 days after middle cerebral artery occlusion (MCAO) or sham operation. Data are presented as mean ± SEM, *n* = 5–6 per group. (D) Temporal changes of erythropoietin receptor (EPOR) and βCR protein expression in the ipsilateral brain tissue detected by western blot. β‐Actin served as a loading control. EPOR (E) and βCR (F) protein levels were quantified at 0.5, 1, 3, 7, and 14 days after MCAO or sham operation. Data are presented as mean ± SEM, *n* = 4–5 per group. *p < 0.05, MCAO 3d, MCAO 7d, or MCAO 14d group vs. Sham group, respectively. (G) Representative immunofluorescence images showing colocalization of EPOR (red) and βCR (green) in the peripheral area of brain infarction. DAPI (blue) indicates the cell nuclei. The white arrows point to the colabeling cells. Scale bar, 100 μm.

The TTC results revealed a significant increase in the percentage of brain infarction volume at 0.5 days and a substantial increase in the percentage of brain edema volume at 1 and 3 days, indicating a gradual increase in the degree of cerebral injury at these time points after MCAO (Figure 6A–C). The western blot results revealed a significant decrease in the βCR protein levels at 3 days after MCAO compared with the sham group (*p* < 0.05, Figure 6D,F). However, there was no evident change in the protein level of EPOR in injured brains at different time points (Figure 6D,E). These western blot results indicated a rapid fall tendency in the βCR protein levels after MCAO surgery, accompanied by increased brain tissue damage (Figure 6F). The immunostaining showed that EPOR/βCR expression in the brain remains very low or absent under normal conditions. Significant activation of EPOR/βCR double‐positive cells was observed in the peripheral area of the brain infarction 1 day after MCAO surgery. EPOR/βCR double‐positive cells significantly decreased at 3 and 7 days following MCAO (Figure 6G). Consistent with the western blot result, the number of EPOR‐positive cells did not vary significantly at each observation time point; in contrast, the number of βCR‐positive cells decreased.

ARA290, also known as pyroglutamate helix B surface peptide, reportedly exhibits cytoprotective activity in various models, including myocardial, renal ischemia‐reperfusion, traumatic brain injury, and others, when administered at doses ranging from 0.5 to 100 μg/kg.^16^ Especially in neurological disorders, a previous study indicated ARA290 (30 μg/kg, bid) exhibited neuroprotective activities comparable to those of EPO in mild traumatic brain injury (mTBI) followed by hemorrhagic shock.^18^ Robertson et al. also found that ARA290 (30 μg/kg, bid) improved Morris water maze performance and reduced inflammatory cells in the cortex of a mice model of mTBI.^19^ Chen et al. demonstrated that ARA290 (35 and 70 μg/kg, qd) suppressed inflammation in experimental autoimmune encephalomyelitis in Lewis rats.^20^ Compared to the findings of the studies mentioned above, different concentrations and administration frequencies of ARA290 were employed in the treatment of cerebral ischemic mice in this study. The results indicated that administration of ARA290 at 30 μg/kg twice daily exerted a significant neuroprotective effect. TTC analysis of brain sections in different groups revealed that a single daily injection of ARA290 (30 μg/kg, or 100 μg/kg once daily) did not prevent brain tissue damage 7 days after cerebral ischemic stroke. This outcome may be attributed to the very short plasma half‐life of ARA290 (approximately 2 min) in circulation.^9^

To our knowledge, no published studies have explored the effects of EPO treatment on the spleen after a cerebral ischemic stroke. In this study, there was a significant increase in both the weight and length of the spleen in EPO‐treated mice. EPO treatment also significantly increased erythropoietic parameters, including the RDW‐CV, RDW‐SD, and MCV. These results indicate a substantial increase in abnormal erythrocytes in EPO‐treated mice, and the increased number of these abnormal erythrocytes in circulation may cause massive hypersplenism and splenic engorgement (splenomegaly). The spleen is the primary site of extramedullary hematopoiesis in pathological conditions, making it prone to engorgement owing to cell proliferation. Whether splenomegaly is transient (the spleen returns to normal size upon recovery) or persistent, it can compress the surrounding organs, leading to abdominal discomfort, pain, and diaphragmatic irritation. These factors can impact the disease prognosis.^21^ As anticipated, this study revealed no significant changes in peripheral blood erythropoietic parameters, serum AST, ALT, LDH, and spleen weight and length after ARA290 treatment. As mentioned earlier, the anti‐apoptotic and anti‐inflammatory activities of ARA290 have been confirmed by previous studies in various models,^22^, ^23^ and our results further demonstrated that these ARA290 effects extend to cerebral ischemic models. Untargeted LC‐MS/MS analyses revealed that the primary differential metabolites affected by ARA290 were associated with the pathophysiology of the vascular endothelium, which may be related to its anti‐apoptotic and anti‐inflammatory properties. This is because research has demonstrated that apoptosis, inflammation, and oxidative stress play a central role in the pathophysiology of the vascular endothelium. Consequently, this study verified the safety and efficacy of ARA290 as a neuroprotective agent. In the past 20 years, several clinical trials have suggested that EPO is unlikely to be a suitable drug candidate for neuroprotection due to its hematopoietic side effects. ARA290 mediates the positive responses of EPO while avoiding the adverse effects.

Conflicting reports exist regarding the role of EPOR/βCR or βCR in mediating tissue‐protective signals in response to EPO and its derivatives. This study aimed to address this issue. Previous studies identified the critical role of EPOR in the normal development of a mouse; the knockout of EPOR in a mouse fetus resulted in the mouse's death.^24^, ^25^ In our preliminary experiment, we also inhibited the expression of EPOR by intracerebroventricular injection of siRNA against EPOR to investigate its role in the neuroprotective effect. However, a high mortality rate (approximately 60%) appeared in the MCAO + EPOR siRNA group. Therefore, increasing evidence suggests that EPOR siRNA gene silencing is more suitable for a series of in vitro cell studies than in vivo animal model studies. According to the above reasons, we inhibited the expression of βCR alone by intracerebroventricular injection of siRNA against βCR to investigate its role in brain tissue protection. Our data demonstrated that the neuroprotective effects of ARA290 and EPO were significantly suppressed by βCR siRNA treatment, indicating the necessity of βCR for ARA290 and EPO to exert their neuroprotective effects. Although this study did not investigate the direct interaction between the two receptors, our data indicated that EPOR and βCR could colocate in the same cytoplasm in the peripheral area of brain infarction following a cerebral ischemic insult. This finding is consistent with several studies that reported the presence or upregulation of EPOR and βCR during tissue injury induced in animal models or cells.^11^, ^12^, ^25^ Our research revealed a significant decrease in the βCR protein level and the number of βCR‐positive cells 3 days after cerebral ischemic stroke. This trend continued until the end of the study (14 days after MCAO). However, there was no significant change in the protein level of EPOR or the number of EPOR‐positive cells in the injured brains at different time points. The degree of tissue damage also increased with prolonged cerebral perfusion, as demonstrated by the increasing cerebral infarction volume 1 and 3 days after MCAO and the increasing brain tissue loss volume at 7 and 14 days after MCAO. Combined with our findings and previous research results, we can speculate that the unsatisfactory therapeutic effects of delayed neuroprotectant administration in ischemic stroke patients, within the short time window for effective treatment may be related to the loss of βCR expression in the early stages of stroke.

## 4 CONCLUSION

Our study had some limitations. First, in this experiment, we did not demonstrate whether the enhanced expression of βCR or EPOR significantly enhanced the neuroprotective effects of ARA290 or EPO. Second, the causal relationship between EPOR and the neuroprotective effect of ARA290 has not yet been fully defined. Therefore, more stereoscopic research should be carried out to explore the potential effect of upstream genes on βCR regulation or EPOR/βCR formation to enhance protection. Nevertheless, this study is the first to demonstrate that ARA290 can inhibit neuronal apoptosis and inflammatory reactions via βCR following a cerebral ischemic stroke. ARA290 did not remarkably affect the weight and length of the spleen or erythropoietic parameters in the peripheral blood. Therefore, ARA290 is a promising therapeutic candidate for the treatment of cerebral ischemic stroke.

## AUTHOR CONTRIBUTIONS

Z.Y., Y.H., and F.Y. performed the animal experiments and analyzed the data. R.W., Y.H., and Y.W. performed molecular biology experiments and analyzed the data. R.W., Y.Z., Z.H., J.F., and Z.T. discussed the results and contributed to the manuscript preparation. R.W. and H.Z. wrote the manuscript. S.L. and Y.L. designed and supervised the project. All authors have read and approved the final manuscript.

## CONFLICT OF INTEREST STATEMENT

<Luo, Yumin> is an Editorial Board member of CNS Neuroscience and Therapeutics and a corresponding author of this article.

## Data Availability

The data that support the findings of this study are available from the corresponding author upon reasonable request.
